# Sperm allocation in relation to female size in a semelparous salmonid

**DOI:** 10.1098/rsos.160497

**Published:** 2016-12-14

**Authors:** Yuya Makiguchi, Masaki Ichimura, Takenori Kitayama, Yuuki Kawabata, Takashi Kitagawa, Takahito Kojima, Trevor E. Pitcher

**Affiliations:** 1 College of Bioresource Sciences, Nihon University, 1866 Kameino, Fujisawa, Kanagawa 252-0880, Japan; 2 Shibetsu Salmon Museum, Shibetsu, Hokkaido 086-1631, Japan; 3 Institute for East China Sea Research, Nagasaki University, Nagasaki 851-2213, Japan; 4 Atmosphere and Ocean Research Institute, The University of Tokyo, 5-1-5 Kashiwanoha, Kashiwa, Chiba 277-8564, Japan; 5 Department of Biological Sciences, University of Windsor, Windsor, Ontario, CanadaN9B 3P4; 6 Great Lakes Institute for Environmental Research, University of Windsor, Windsor, Ontario, CanadaN9B 3P4

**Keywords:** spawning behaviour, mate choice, acceleration data loggers, female body size

## Abstract

To maximize reproductive success, males have to adaptively tailor their sperm expenditure in relation to the quality of potential mates because they require time to replenish their sperm supply for subsequent mating opportunities. Therefore, in mating contexts where males must choose among females in a short period of time, as is the case with semelparous species (which die after one intensely competitive short duration breeding season), selection on sperm allocation can be expected to be a powerful selective agent that shapes the male reproductive success. We quantitatively investigated sperm allocation patterns in chum salmon in relation to perceived female quality by developing a novel method for determining the amount of sperm allocated per ejaculate during spawning bouts. We examined the relationship between sperm expenditure and the body size of paired females (a proxy of egg number and egg quality) in the absence of male–male competition in an experimental channel. The estimated amount of sperm released per spawning event was positively correlated with the size of paired females. However, the number of spawning events a female participated in, which reduces the number of eggs she spawns in each subsequent bout, did not affect this relationship. These results provide support for predictions arising from the sperm allocation hypothesis, male salmon do economize their sperm expenditure in accordance with paired female body size as predicted for their first spawning event, but males overestimate or are unable to assess the quality of females beyond size and provide more sperm than they should in theory when paired with a female that spawned previously. Overall, the observed sperm allocation pattern in chum salmon appears to be adapted to maximize reproductive success assuming female size is an honest indicator of quality, although temporal changes in a female's quality during a reproductive season should be considered when examining sperm allocation strategies.

## 1. Introduction

An important challenge in evolutionary biology has been to understand strategies through which males invest in sperm production and allocation of sperm among mating opportunities to maximize reproductive success [1]. A large number of studies in the last two decades have shown that sperm production can be limited, energetically costly and slow to replenish under certain circumstances [2,3]. Therefore, males should in theory tailor their sperm storage and expenditure in response to mating opportunities and female quality (e.g. fecundity) to maximize their reproductive success [4–7]. For example, male fowl (*Gallus gallus*) preferentially allocate sperm to females with large sexual ornaments which presumably signal superior maternal investment [8]. Males can also face another challenge with respect to sperm allocation: there are limitations in sperm reserves for immediate use in reproduction, thus males often cannot consecutively mate with females (e.g. [9]). Sperm reserves can be depleted over successive mating opportunities and may result in the lack of sufficient storage of sperm to fertilize all possible eggs [10]. Males typically require time to replenish sperm and serum reserves between mating attempts, suggesting that there is a relationship between the duration of breeding seasons and sperm allocation in terms of the reproductive success of males. For example, sperm allocation between mating attempts in males is expected to affect reproductive success if the breeding season is very limited or only one season is available to reproduce. However, previous empirical studies on sperm allocation have not focused on species possessing life histories with only one short breeding season with intensified reproductive competition among males, such as a semelparous life history (but see [11]).

Chum salmon (*Oncorhynchus keta*) is a semelparous and external fertilizing salmonid. As the breeding season approaches, sexually mature individuals migrate to natal streams from the sea in order to spawn [12,13]. On the spawning grounds, females select spawning sites and construct nests by digging and displacing substrate with their tails. Simultaneously, males compete among each other for access to females and mate with females under intense male–male competition [14,15]. Dominant males vibrate their body closely behind the females (quivering behaviour) and cross over the females, which encourages nest construction by females [12,16]. After the oviposition is complete, females guard their nests to avoid having their eggs uncovered by other females (rather than buried). All individuals die within a few weeks of the beginning of the spawning period, which means only one breeding season per individual [17].

Many studies on mate choice (reviewed in [18,19]), including in salmonids [14,20], have pointed out the importance of adult body size. Previous studies on female choice in salmonids showed that females delayed spawning behaviour in the presence of relatively small males in chum salmon, sockeye salmon, *O. nerka* and chinook salmon, *O. tshawytscha* [21–24]. These results have been recognized as an indirect form of female mate choice. On the other hand, there are only a few studies that show evidence of male mate choice for body size in salmonids. Foote [25] found that males prefer the largest female, which resulted from a correlation between female size and male aggregation size on the spawning ground. Males may benefit from choosing larger females because they produce larger eggs, monopolize higher quality spawning ground for egg development, and also dig deeper redds and defend their nests longer against nest construction by the other females [20,26,27], which may help to increase the reproductive success. Males spend more time and energy searching for mating opportunities on the spawning ground than females [28,29]. Therefore, male salmon can be faced with a conflicting demand of investing in costs related to searching for larger females or mating with nearby females. Semelparous male salmon are, therefore, predicted to allocate sperm per mating opportunity so as to maximize reproductive success in the very limited and intense breeding season. In salmon, where females vary in reproductive value based on size (larger females typically have more and higher quality eggs [30,31]), males are predicted to strategically allocate their sperm in relation to female size to maximize their reproductive success [8].

In this study, we predict that male chum salmon allocate their sperm in relation to female size; more sperm will be allocated to relatively and absolutely larger females in the one male–one female situation. This hypothesis has not been examined in salmonids previously because of the difficulty in determining sperm expenditure during spawning events. The reason for this difficulty is that released milt (sperm and seminal plasma) is rapidly diluted due to the high speed currents of streams and rivers following ejaculation. To resolve this issue, we developed a sophisticated method to measure the amount of sperm ejaculated per spawning bout using a modified method originally conceived by Fitzpatrick & Liley [32]. Specifically, to attach the condom device, 8–10 stiches are made around the abdominal cavity. In addition, we used animal-borne data loggers to record acceleration based data, including vibrations of the trunk musculature at the moment of gamete release [33], which we show correlates with sperm volume expended by males during individual spawning bouts. The overall objective of this study was to examine the sperm allocation and spawning behaviour of males in relation to female quality (i.e. variation in size) under non-competitive semi-natural spawning conditions. We also examined the size–fecundity relationship in females of this species in order to examine the assumption that female size is a good surrogate for egg quality and number deposited per spawning bout.

## 2. Material and methods

### 2.1. Fish collection

Sexually mature male and female chum salmon were collected in November of 2011 (seven males and seven females), 2012 (11 males and 11 females) and 2013 (21 males and 21 females) using an ‘Urai’ trap set in the Shibetsu River estuary (43°43′ N, 145°7′ E, Hokkaido, Japan). The fish were transported to an outdoor tank separately and separated by sex at the Shibetsu Salmon Museum, Hokkaido, Japan until the experiment began. The fish used in this study were considered virgins (as they had not yet spawned) at the beginning of the experiment. While being collected for tagging (see below), all fish were confirmed to be spermiating (i.e. sperm released under gentle pressure on the abdomen) or gravid (egg expressed during abdominal massage).

### 2.2. Collection of milt

**Figure 1.**
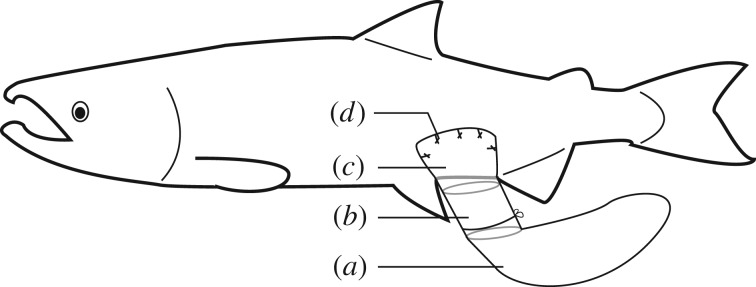
Condom (*a*) is connected to 17 mm diameter vinyl tube (*b*) and piece of glove (*c*). The vinyl tube was used to prevent the compression of the abdominal cavity during gamete release. To attach the condom device, 8–10 stiches (*d*) are made around the abdominal cavity. This attachment procedure took 25 min to complete.

Collection of milt ejaculated at the moment of gamete release was achieved using a latex condom attached to each male according to methods adapted from a previous study [32]. A 400 mm piece of 17 mm diameter vinyl tube with a piece of latex glove was tied tightly to a latex condom. The condom device was surgically attached by using eight to 10 sutures to cover the abdominal area of the experimental fish under anaesthesia, which prevented the compression of the abdominal cavity during gamete release after the tagging procedure of the data loggers described above (see figure 1 for the condom device used for collecting sperm). Immediately prior to the attachment of the condom, 100 ml of river water was poured into the condom, which assured that the condom remained open during spawning to allow collection of the released sperm. The surgical procedure took 25 min to complete per individual. During the procedure, the gills of the fish were irrigated with water containing a 0.25 ml l^−1^ concentration of FA 100 (eugenol; Tanabe Seiyaku Co. Ltd, Osaka, Japan) to maintain sedation. After the procedure was completed, the fish was moved to the spawning channel, where the spawning behaviour was monitored by at least two observers through a glass window in the spawning channel with a digital video camera. Immediately after gamete release, males were removed from the spawning channel and the condom was detached from the tube, pinching the neck of the condom to prevent leakage. The total amount of diluted released sperm was measured using a graduated cylinder. The concentration of sperm in milt was determined using a spectrophotometer (PD-303, APEL Co., Ltd., Saitama, Japan) at a wavelength of 410 nm [32]. A calibration curve was developed using milt collected from males before the experiments started to estimate the amount of released sperm for each individual. Sperm obtained under gentle pressure on the abdomen was diluted at 0.5, 1.0, 2.0, 4.0, 8.0, 16.0 and 32.0 µl ml^−1^ with river water and the absorbance at a wavelength of 410 nm was measured using the spectrophotometer. The relationship between absorbance values and sperm concentrations at each diluted concentration was described using exponential function and was plotted to create a standard curve for each concentration for each individual. Each sample was measured in duplicate in the spectrophotometer and the absorbance values were averaged. The total amount of sperm released was calculated by multiplying the concentration of sperm released by the total amount of diluted sperm. The absorbance of known diluted sperm was significantly and positively correlated with amount of sperm (electronic supplementary material, figure S1, *p* < 0.05; concentration of sperm (μl ml^−1^) = 0.65 exp^2.2 Absorbance^, average of eight males *r*^2^ = 0.95 ± 0.02)). The equations from the correlation analysis were individually used to convert absorbance of sperm collected to each male's amount of sperm release. Furthermore a linear mixed model found that the estimated amount of sperm released was significantly and positively correlated with vibration duration at the sperm release, which was estimated as amount of sperm release = e^(0.21 × vibration duration at sperm release (s)+2.59)^; electronic supplementary material, figure S2; table 1).

**Table 1.** Linear mixed model examining the effects of dependent variables on relationships between the amount of sperm released and vibration duration at sperm release. A likelihood ratio test was used to examine the significance of the fixed effects compared with vibration duration at sperm release model. Full model represents model including all variables. Reduced model represents model reducing one variable. For example, reduced model of ‘vibration duration at sperm release’ model means no variable model (null model). *p*-value of the vibration duration at sperm release model is a result of comparison with null model examined by a likelihood ratio test. AIC, Akaike's information criteria; AICc, second-order AIC; CI, 95% confidence interval for a coefficient.

	full model								reduced model		
					CI for a coefficient						
models	AIC	AICc	log-likelihood	coefficient	lower	upper	s.e.	*R*^2^	AIC	log-likelihood	*p*-value
vibration duration at sperm release	−2.65	10.90	5.32	0.21	0.18	0.25	0.02	0.85	32.82	−13.41	<0.001
vibration duration at sperm release + male fork length	−3.47	24.71	6.73	0.21	0.18	0.25	0.02	0.88	−2.65	5.32	0.09
				0.0017	−0.0004	0.0039	0.001				
vibration duration at sperm release + number of sperm release	−0.78	19.16	5.39	0.21	0.18	0.25	0.02	0.86	−2.65	5.32	0.72
				−0.02	−0.13	0.09	0.06				

### 2.3. Data logger attachment

To measure the swaying acceleration profile to estimate vibration duration related to spawning behaviour and ultimately sperm expenditure, the male and female fish were tagged with cylindrical acceleration data loggers (M190 L-D2GT: 15 mm in diameter, 53 mm in length, 18 g in air; Little Leonardo Co., Tokyo, Japan, see [34] for details) that could record two-axis acceleration (surging and swaying) at 1/32 s intervals. The attachment procedures, including the measurements of the mass, girth and fork length of the fish required five minutes to complete per individual.

### 2.4. Vibration measures

**Figure 2.**
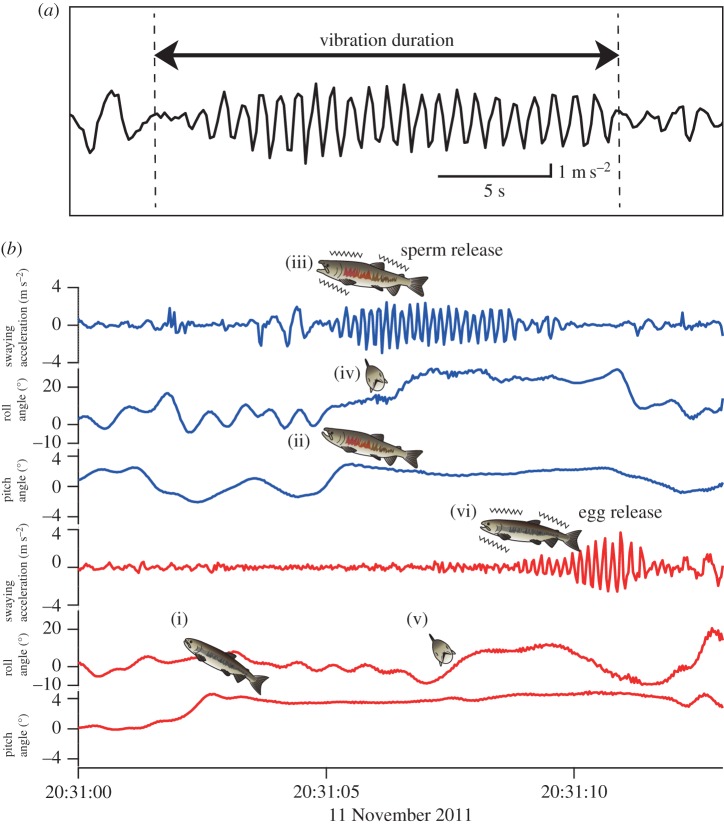
(*a*) Typical swaying acceleration profile at the moment of gamete release in a male showing a characteristic vibration at 7–8 Hz between arrows, which is calculated as vibration duration (seconds). (*b*) Typical profiles for acceleration data during spawning: the blue lines represent the male and the red line represents the female. When the female completes the building of the nesting site (red), the female puts its anal fin into the ground and gapes (i). Then, the male moves to the side of the female (ii) and releases sperm (iii), leaning its body in a sway axis (iv). This vibration pattern causes females to lean its body in a sway axis (v) and release a subset of eggs (vi). There is always a time lag (approx. 2.3 s) between releasing sperm and releasing eggs.

To measure the vibration duration at sperm release, we measured both swaying and surging acceleration data retrieved from tagged fish. The acceleration data loggers measured both dynamic acceleration (contractions of trunk muscular activity) and static acceleration along the body axis (gravity or pitch). To separate these values, a continuous wavelet transform (CWT) filter with Igor Pro (WaveMetrics Inc., Lake Oswego, OR, USA) and Ethographer software [35] was used to extract the information on body vibration at gamete release from swaying acceleration and pitch from surging acceleration, respectively. Duration of gamete release of males was defined as duration of body vibration when oviposition was observed, measured by the swaying acceleration (figure 2). Convulsive contractions of trunk muscular activity corresponds to gamete release, as has been revealed by studies using electromyogram recordings [33,36,37].

### 2.5. Sperm quantity and vibration duration

To examine the relationship between the sperm quantity expelled during spawning and vibration duration, we monitored the spawning behaviour of pairs (one male and one female) in a spawning channel (3.8 × 2.9 × 1.1 m) in the Shibetsu Salmon Museum, Shibetsu, Hokkaido. The spawning channel was supplied with Shibetsu River water as spring water underneath a gravel bottom, which was free of silt [13]. At night, the spawning channels were provided minimal light to record the spawning behaviour. An atomic radio controlled clock (LED-101 BU, Seiji Corporation, Yokohama, Japan) was also installed in front of the spawning channels to synchronize time series and calibrate the spawning behaviour and the acceleration data measured by the data loggers.

To determine the relationship between the vibration duration and the amount of released sperm during spawning behaviour, males were fitted with a condom to collect the ejaculate (see details above) and an acceleration data logger. They were paired with females fitted with acceleration data loggers and were monitored with a digital video camera (GZ-HM670, JVC KENWOOD Corporation, Yokohama, Japan) in the spawning channel until oviposition was observed. One male fitted with a condom to collect the ejaculate and the acceleration data logger and one gravid female were moved to the spawning channel. Once spawning behaviour was observed, we removed the male fish from the channel to collect the ejaculate and attached a new condom on the same fish. In this experiment, eight males (626 ± 62 mm fork length, 2.6 ± 0.9 kg mass) and nine females (589 ± 40 mm fork length, 2.2 ± 0.5 kg mass) were used in total in 2012. We collected more than one sample for six of the males and two females spawned with two different males.

### 2.6. Sperm quantity and female size

The second experiment was done without directly measuring sperm release but rather using the vibration data to estimate it. To examine the relationships between the estimated amount of sperm released during spawning and the relative and absolute female size, we monitored the spawning behaviour of pairs (one male and one female) in a spawning channel in the Shibetsu Salmon Museum, Shibetsu, Hokkaido. To examine whether males regulate the amount of sperm released, the spawning behaviour of males and females with the acceleration data loggers was monitored with the digital video camera in the spawning channel. In this experiment, 39 females (619 ± 48 mm fork length, 2.5 ± 0.6 kg mass) and 39 males (618 ± 62 mm fork length, 2.6 ± 0.9 kg mass) were used. The variation in body size of fish used was not large across years. For example, male fork length is 621.1 ± 26.3 mm in 2011, 596.6 ± 15.1 mm in 2012 and 627.6 ± 13.9 mm in 2013. The relationship between the amount of sperm estimated at gamete release of males and the relative and absolute paired female size to male size were examined in linear mixed models (see Statistical analyses for detail). The data of the estimated amount of sperm was included for the first, second, third, fourth and fifth ovipositions.

### 2.7. Statistical analyses

Linear mixed models were used to examine the relationships between the vibration duration and the amount of sperm released and were also used to examine the associations between the estimated amount of sperm released at spawning, and (i) the relative female size to male size, (ii) male fork length, (iii) female fork length, (iv) mating event number for each male, (v) mating event number for each female. Because we observed gamete release behaviour several times for the same pair, pair identity was fitted as a random factor in the model to avoid pseudoreplication. *F*-test revealed that there was no significant variation in fork length between sexes (*F* = 1.58, d.f. = 38, *p* = 0.165). Linear mixed models were used to examine the relationship between the size (fork length) of females and egg diameter (mm) and egg weight (mg). Generalized linear mixed effects models were used to examine the relationship between the size (fork length) of females and number of eggs using a Poisson distribution. Therefore, the data of number of eggs was log transformed. Year was fitted as a random factor in the model. Generalized linear mixed effects models were also used to examine the relationship between mating event number in females and number of eggs released. Fish identity was fitted as a random factor in the model. Tukey–Kramer HSD tests were used to compare the number of eggs released between matings. For model fitting, we used the software R v. 3.1.1 [38] with the lmer function in R packages lme4 and multcomp, and 95% CI values for slope were obtained to simulate 10 000 response vectors via the bootstrapping method. A likelihood ratio test was used to assess changes in fitted model compared with reduced model and *p*-values for each parameter. Statistical significance was achieved when *p* < 0.05; values are presented as mean ± s.d.

## 3. Results

### 3.1. Amount of ejaculated sperm and trunk vibration duration

All tagged males spawned either once or twice. In the experiment, we changed a female between the first and second oviposition for a male, therefore there was a difference in number of fish we used between sexes. Sixteen instances of sperm ejaculation in males were observed and sixteen sperm samples with the acceleration data at the sperm release were collected in total (see electronic supplementary material, movie S1, for an example of oviposition, in which a male was attached with the device for collecting sperm). Figure 2*a* shows a representative swaying acceleration profile at the moment of sperm release for a single male; trunk vibration duration at sperm release was measured from the swaying acceleration profiles. Linear mixed models found that there was a significant relationship between the estimated amount of sperm released and vibration duration at the time of sperm release (electronic supplementary material, figure S2; table 1). Male fork length and mating event number males participated in did not significantly affect the relationship (table 1); however, AIC of model including vibration duration and male fork length was lowest in these models. Therefore, we used the model including vibration duration and male fork length to estimate the amount of sperm released.

### 3.2. Estimated amount of ejaculated sperm and paired female size

**Figure 3.**
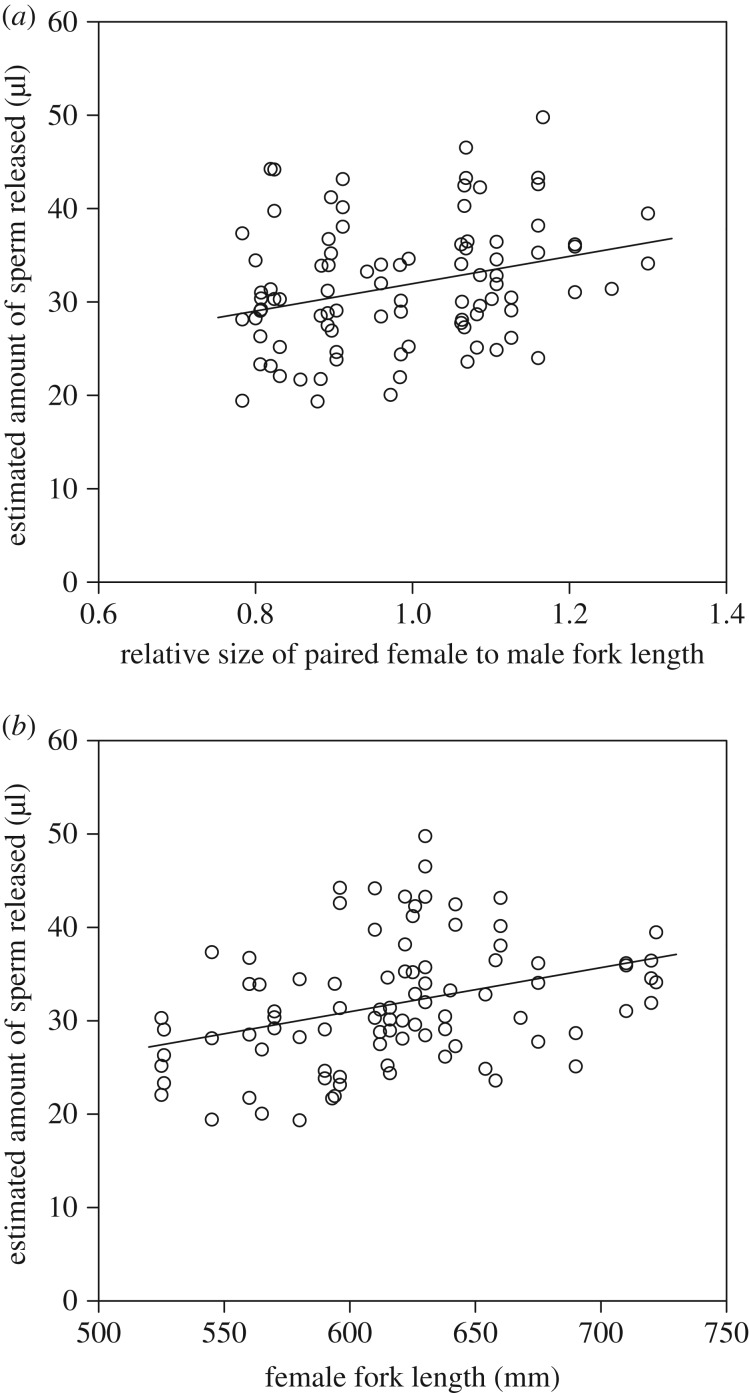
(*a*) Relationship between the amount of sperm released and the relative size of paired female to male fork length. The curve was fitted by: amount of sperm release = 37.90 × relative size of paired female to male fork length. (*b*) Relationship between the amount of sperm released and female fork length. The curve was fitted by: amount of sperm release = 0.068 × female fork length – 9.64. Data were obtained from 90 ovipositions for 39 males and 39 females.

All tagged fish (39 males and 39 females) spawned at least once and at most five times. Ninety instances of oviposition were observed, and the acceleration data for analysis were measured for all of the oviposition events. Figure 2*b* shows a typical representation of the swaying acceleration calculated from the surging acceleration profiles at the moment of gamete release in paired fish. Linear mixed models found that there was a significant positive correlation between the amount of sperm estimated and the relative size of paired female to male fork length (figure 3*a*) as well as the female fork length (figure 3*b*, table 2). There was no significant correlation between the amount of sperm estimated and the male fork length (table 2; electronic supplementary material, figure S3). In addition, there was no significant correlation between the amount of sperm estimated and mating event number in males and females (table 2). To investigate the effects of duration between matings in females on the amount of sperm released, we also used models containing both female fork length and log-transformed duration between matings (data from 31 females that spawned more than twice) to explain the amount of sperm released using linear mixed models (electronic supplementary material, table S1), and found that female fork length and log-transformed duration between matings positively correlated with the amount of sperm released (electronic supplementary material, figure S4). To investigate effects of duration between matings in females, we compared the models containing female fork length, male fork length or the relative female size to paired male size with the null model using linear mixed models (electronic supplementary material, table S2). The parameters did not significantly affect the duration between matings except for the amount of sperm released.

**Table 2.** Linear mixed model examining the effects of dependent variables on the estimated amount of sperm released. A likelihood ratio test was used to the significance of the fixed effects compared with null model. AIC, Akaike's information criteria; AICc, second-order AIC; CI, 95% confidence interval for a coefficient.

	estimates							
					CI for a coefficient			
dependent variables	AIC	AICc	log likelihood	coefficient	lower	upper	s.e.	*p*-value
null	599.2	622.6	−295.8	—	—	—	—	—
female/male size ratio	595.0	592.1	−290.1	14.63	3.15	26.27	5.88	0.01
male fork length	600.5	613.0	−298.9	−0.011	−0.039	0.017	0.014	0.42
female fork length	591.6	619.6	−294.4	0.047	0.018	0.077	0.015	<0.01
mating event number in males	600.9	609.8	−295.1	0.412	−1.073	1.920	0.745	0.58
mating event number in females	598.9	619.6	294.0	1.195	−0.396	2.773	0.806	0.14

### 3.3. Female size and gamete quality

**Figure 4.**
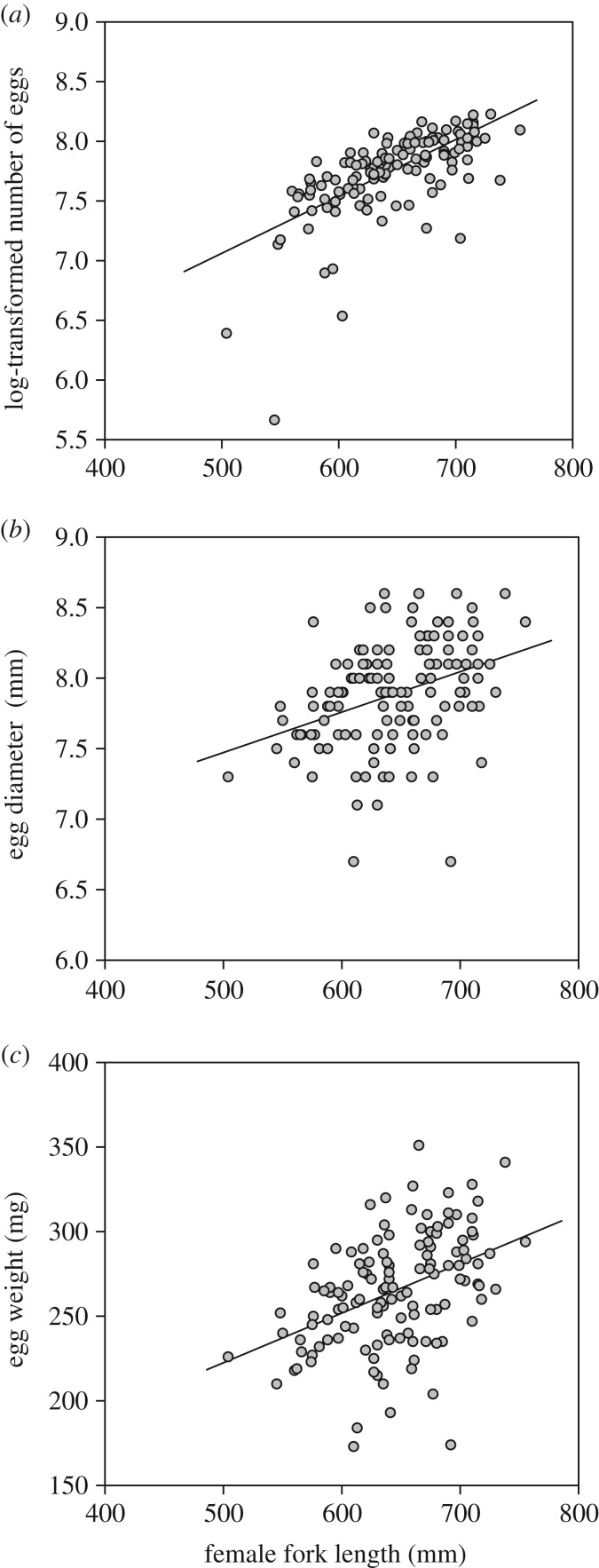
Relationship between female fork length (mm), log-transformed number of eggs per a female (*a*), egg diameter (*b*) and egg weight (*c*; *n* = 154): log-transformed number of eggs = 0.005 × female fork length + 4.68, egg diameter (mm) = 6.04 × female fork length + 0.003 and egg weight (mg) = 0.29 × female fork length + 75.68.

A generalized linear mixed model found that there was a significant positive relationship between number of eggs per spawning and female fork length (figure 4*a*). In addition, a linear mixed model found that there was a significant positive relationship between egg diameter or egg weight and female fork length (figure 4*b*,*c*; electronic supplementary material, table S3).

### 3.4. Mating events and number of eggs released

**Figure 5.**
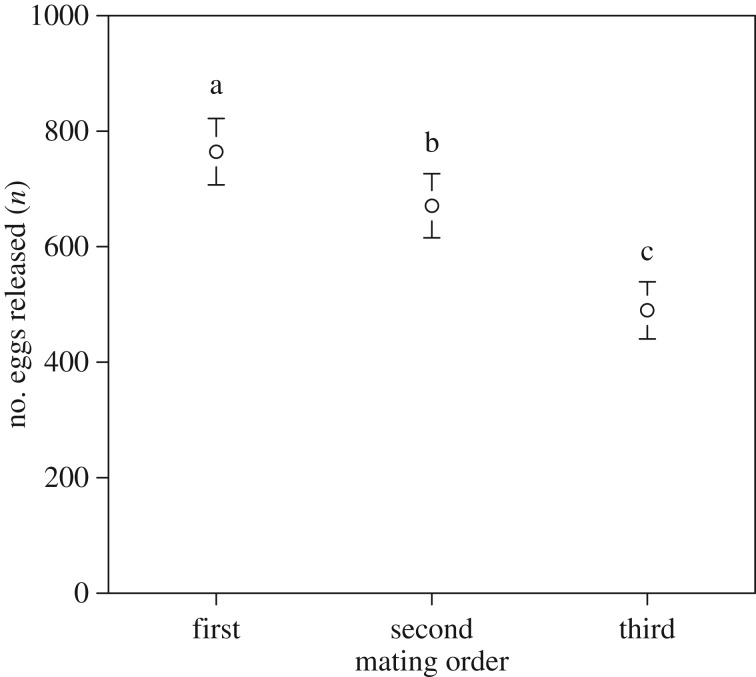
Relationship between the number of eggs released and the mating order in females. Open circles without a common letter differed significantly (*p* < 0.05, see text for details). Data are obtained from 26 females and presented as means ± s.d.

A generalized linear mixed model found that the number of eggs released during a spawning event significantly decreased with an increasing mating event number in females (figure 5). Therefore, we examined the relationship between log-transformed number of eggs and female fork length each mating, respectively. A generalized linear mixed model found that there was a significant positive correlation between log-transformed numbers of eggs and female fork length in each mating event number (electronic supplementary material, figure S5).

## 4. Discussion

We found positive relationships between the amounts of sperm ejaculated per spawning bout and the relative size of the paired female, thus providing evidence that male salmon adjust sperm ejaculate allocation according to perceived female quality. This result is probably due to the strong relationship between female size and fecundity in salmonid species [25,39], including our population of chum salmon. We also found a positive relationship between female size and number of eggs, egg diameter and egg weight, and number of eggs released per spawning event. A previous study reported that offspring (i.e. alevins) hatching from large sized eggs (egg diameter and egg weight) had greater amounts of yolk and were bigger compared with those individuals hatching from smaller eggs [40]. In other words, larger females can produce offspring with more yolk reserves and more body tissue [31], which suggests offspring produced from larger females have higher survival rates than those from smaller females [41,42]. In general, larger (that are assumed to be of higher quality) females are allocated more sperm per mating event in several animal species [5,43–46], including fish [2,47].

We interpret our results that males adjust the amount of sperm released per spawning event based on female size as a strategic investment in sperm allocation in chum salmon. In all semelparous pacific salmon (including the focal species), the breeding season is accomplished in a few days or weeks during which time energy resources are rapidly diminished [48]. Our data raise two possibilities with respect to the strategic allocation of ejaculate size in male chum salmon. First, chum salmon may economize their sperm according to the female size due to costly sperm production. However, this possibility seems unlikely because the relative testes mass (gonad mass/body mass) was 1.2% at the end of the spawning season, which decreased from 3.1% at the start of the spawning season [49], and the majority of the testes was still remaining in dead male chum salmon (Y. Makiguchi 2011, unpublished data). Second, the strategic investment in ejaculate size may contribute to maximize the mating opportunities with females, as seen in several taxa (see [18,50]). For semelparous salmon, the loss of mating opportunities could affect their reproductive success due to the restricted mating season. If males spawn rapid consecutive ejaculates, they may face a shortage of sperm to fully fertilize all available eggs, which could result in the loss of mating opportunities because males require time to replenish the ejaculate supplies [10]. As such, it could be that males are better served to select accessible and proximate females as a mate rather than expending time and energy seeking out the largest available females [25].

Previous studies on sperm allocation predicts that adequate sperm ejaculate depends on male condition such as amount of sperm retention and varies with the time course of reproduction, metrics of female quality and frequency of mating [7]. Our study showed that the number of eggs females spawn per mating event significantly decreased with each successive mating event, indicating that female quality (in terms of egg quantity) deceased with each successive mating event. Female abdomens clearly shrank after each successive spawning event (M Ichimura *et al.* 2011 personal observation), because all fish used in this study were virgins. However, the allocation of sperm did not differ between matings; males provided sperm in an optimal manner to the paired females based solely on generic body size and not mating history, indicating that males overestimate female quality (as they continue successive spawning bouts) and apparently cannot assess fine-scale changes in female quality beyond generic body size differences. These results can be attributed to a possible trade-off between the cost of searching for mating opportunities and the cost of sperm production in a very protracted breeding season common to semelparous species. Fleming [20] pointed out that male salmon invest considerable time and energy acquiring females. Therefore, semelparous males might not allocate their sperm optimally if they have the option for possible fertilization opportunities in the near future. We also assumed that the extra sperm allocation can be related to the replenishment of sperm supplies. Our study found positive correlations between the amount of sperm released and the duration of time between matings, suggesting that the duration from last mating positively affected the amount of sperm released. These results indicate that replenishment of sperm could also affect the optimal sperm allocation, but we could not clearly demonstrate the effects within the scope of the present study. Because our experimental design did not allow males to search for other females after oviposition, we could not examine how males behave if the replenishment of their sperm supply is not fully completed.

Finally, sperm allocation also may vary according to the intensity of sperm competition, which is predicted in theoretical studies [7] and supported in empirical studies [44,51], including in fishes [47,52]. Competition between males increases the opportunity for selection nearly an order of magnitude greater than in females (e.g. [15]). Furthermore, on the spawning grounds, the small male salmon sometimes adopt sneaking tactics in order to gain access to spawning females [15]. As such, it is likely that under wild conditions there are potential rival males in the vicinity that they must fight in order to have access to nesting females [13,16], and males are invariably under potential sperm competition in natural spawning grounds even after dominating females. Although we did not allow for sperm competition in this study, we speculate that males also may regulate sperm ejaculate according to the intensity of sperm competition (see [53]), which could be tested using our experiment protocol and design.

In conclusion, we showed that male salmon adjusted the amount of their sperm ejaculate to the relative size of paired females, in response to the relative female size, which is strongly correlated with the number of eggs (fecundity) and egg quality (egg diameter and egg weight); however, sperm allocation did not change (i.e. decrease) in spite of the fact that female fecundity decreases with each successive spawning event. Thus, our results indicate sexual conflict probably exist in this mating system; males attempt to economize their sperm for future mating events, whereas females seek to make sure that their entire complement of eggs are fertilized. Furthermore, we emphasize that male salmon allocate their sperm to females based on general body size related metrics despite the fact that the reproductive quality of females decreases with each successive spawning event, which may suggest that male salmon face a trade-off between searching for new mating opportunities with novel females and economizing the use of costly sperm due to their highly protracted single spawning season.

## Supplementary Material

Supporting information SI Materials and MethodsNumber of eggs and the size of femaleTo examine the relationship between the female size and number of egg, 154 fully matured female chum salmon (611 ± 35 mm fork length (LF)) collected in the Shibetsu River, Japan was used during 2009–2011. Fork length, body mass, number of eggs, and egg diameter and weight (n = 10 eggs per females) were recorded for each female. Counting number of eggs releasedTo counting the number of eggs released each spawning, twenty six fully matured female chum salmon (649 ± 47 mm fork length (LF)) collected in the Shibetsu River, Japan was used during 2003–2009. The spawning behavior of one female and one male was monitored in the spawning channel, and the positions of the nests were recorded. After the spawning behavior was completed, we dug up the nest and counted the　number of eggs each nest.Fig S1. An example of a relationship between absorbance values and sperm concentrations diluted at 0.5, 1.0, 2.0 4.0, 8.0, 16.0, and 32.0 μl /ml with river water for fish c_48 (r 2 = 0.97, p < 0.05). Fig S2. Relationship between vibration duration at sperm release and amount of sperm released collected by the condoms (16 ovipositions for 8 males and 9 females). The curve was fitted by: amount of sperm release = e(0.21×vibration duration at sperm release (sec)+ 2.59) using linear mixed models and p < 0.001 was estimated by using a likelihood ratio test compared with null model.Fig S3. Relationship between the amount of sperm released and male fork length. Data was obtained from 90 ovipositions for 39 males and 39 females.Fig S4. Relationship between the amount of sperm released and the log transformed duration between matings. The curve was fitted by: amount of sperm released = 14.57 × female fork length – 4.28 and p < 0.001 was estimated by using a likelihood ratio test compared with the null model. Data was obtained from 51 ovipositions for 31 males and 31 females.Fig S5. Relationship between the log transformed number of eggs released and the number of matings in females for first mating (a), second mating (b) and third mating (c). The curve was fitted by: log transformed number of eggs = 0.0039×female fork length +4.22 for first mating (95 % confidence interval (CI) for a coefficient ranging from 0.0035 to 0.0043 , p < 0.01: a), log transformed number of eggs = 0.0044×female fork length +3.82 for second mating (95 % CI for a coefficient ranging from 0.0040 to 0.0048 , p < 0.001: b), log transformed number of eggs = 0.0037×female fork length +3.93 for third mating (95 % CI for a coefficient ranging from 0.0032 to 0.0042, p < 0.001: c), respectively.

## Data accessibility

Data are available from the Dryad Digital Repository (http://dx.doi.org/10.5061/dryad.p11s3) [54]
