# Supplementary material for: Sperm allocation in relation to female size in a semelparous salmonid
Source: R Soc Open Sci. 2016 Dec 14;3(12):160497. doi: 10.1098/rsos.160497 (PMC5210678; doi:10.1098/rsos.160497)
Supplement: Supporting information SI Materials and MethodsNumber of eggs and the size of femaleTo examine the relationship between the female size and number of egg, 154 fully matured female chum salmon (611 ± 35 mm fork length (LF)) collected in the Shibetsu River, Japan was used during 2009–2011. Fork length [file rsos160497supp1.docx]

**Supporting information**

**SI Materials and Methods**

**Number of eggs and the size of female**

To examine the relationship between the female size and number of egg, 154 fully matured female chum salmon (611 ± 35 mm fork length (*L*_F_)) collected in the Shibetsu River, Japan was used during 2009-2011. Fork length, body mass, number of eggs, and egg diameter and weight (n = 10 eggs per females) were recorded for each female.

**Counting number of eggs released**

To counting the number of eggs released each spawning, twenty six fully matured female chum salmon (649 ± 47 mm fork length (*L*_F_)) collected in the Shibetsu River, Japan was used during 2003-2009. The spawning behavior of one female and one male was monitored in the spawning channel, and the positions of the nests were recorded. After the spawning behavior was completed, we dug up the nest and counted the　number of eggs each nest.

Fig S1


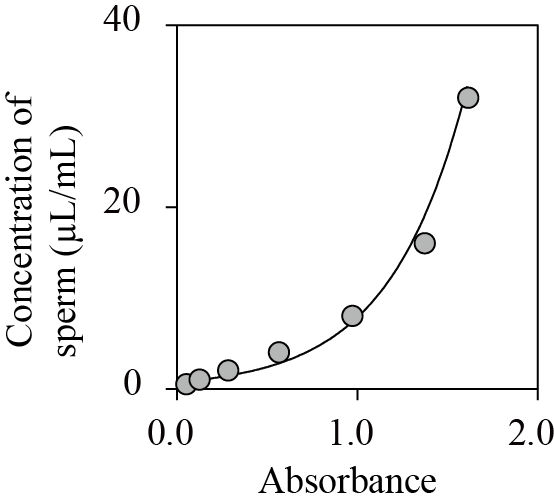


Fig S1. An example of a relationship between absorbance values and sperm concentrations diluted at 0.5, 1.0, 2.0 4.0, 8.0, 16.0, and 32.0 μl /ml with river water for fish c_48 (r ^2^= 0.97, *p* < 0.05).

Fig S2.


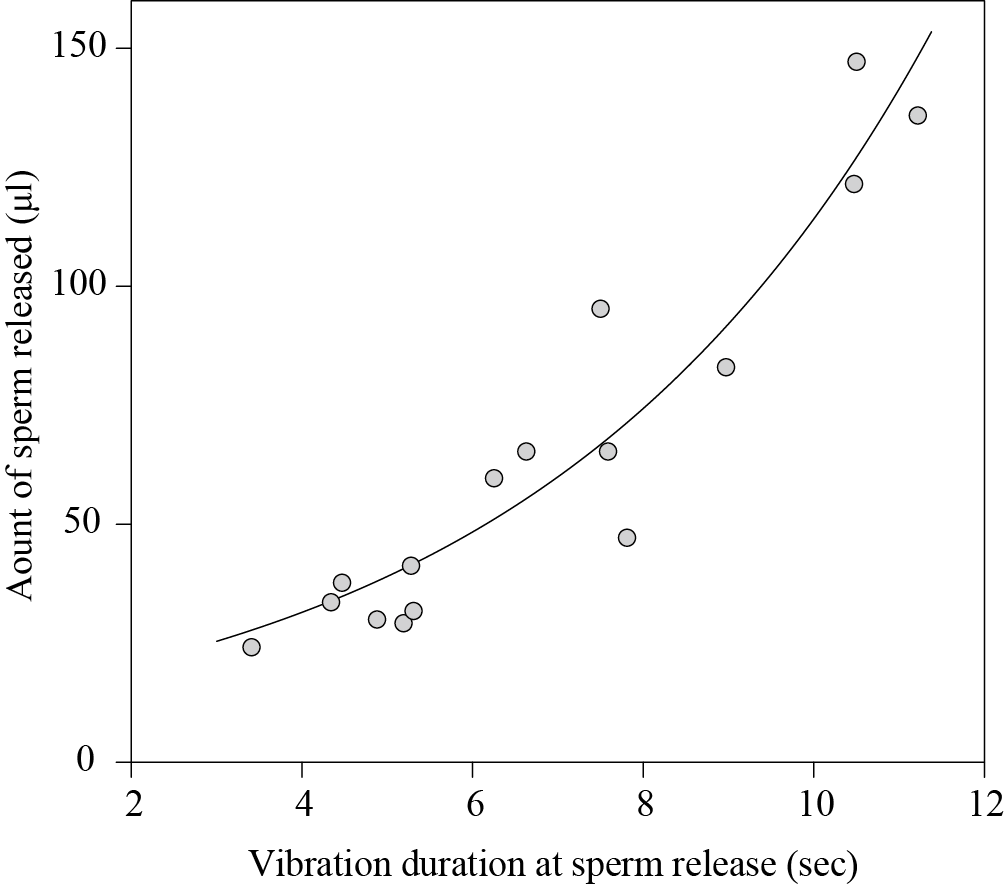


Fig S2. Relationship between vibration duration at sperm release and amount of sperm released collected by the condoms (16 ovipositions for 8 males and 9 females). The curve was fitted by: amount of sperm release = e^(0.21×vibration duration at sperm release (sec)+ 2.59)^ using linear mixed models and *p* < 0.001 was estimated by using a likelihood ratio test compared with null model.

Fig S3.


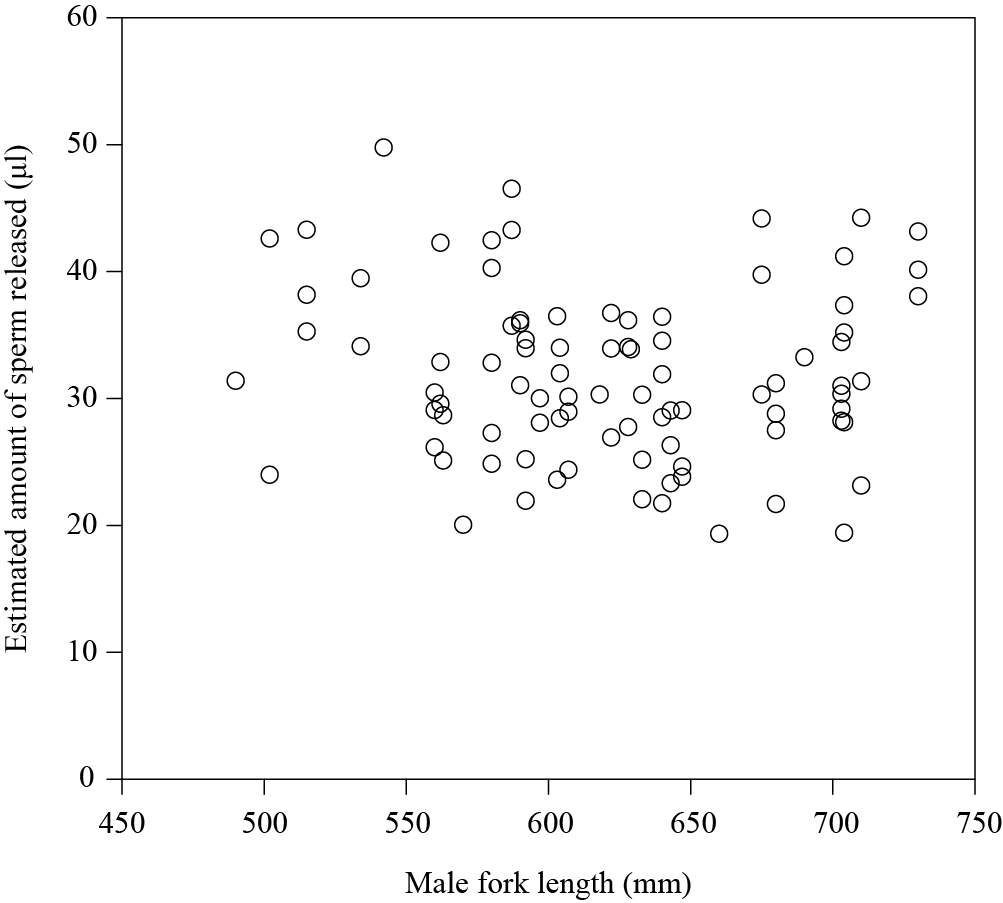


Fig S3. Relationship between the amount of sperm released and male fork length. Data was obtained from 90 ovipositions for 39 males and 39 females.

Fig S4.


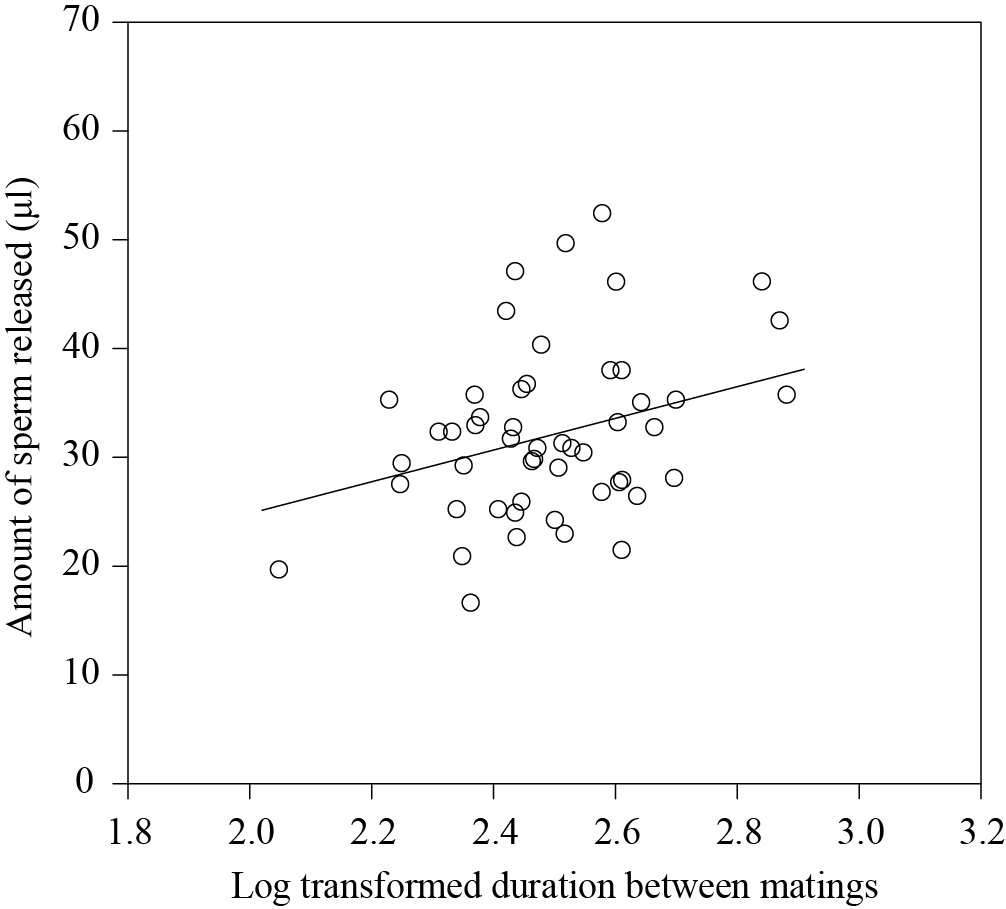


Fig S4. Relationship between the amount of sperm released and the log transformed duration between matings. The curve was fitted by: amount of sperm released = 14.57 × female fork length – 4.28 and *p* < 0.001 was estimated by using a likelihood ratio test compared with the null model. Data was obtained from 51 ovipositions for 31 males and 31 females.

Fig S5.


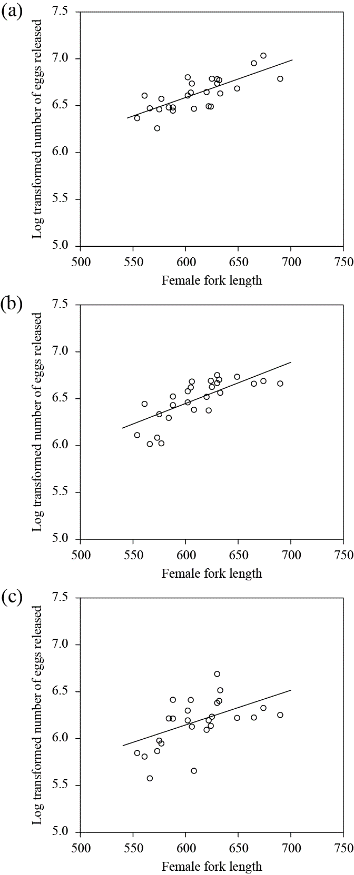


Fig S5. Relationship between the log transformed number of eggs released and the number of matings in females for first mating (a), second mating (b) and third mating (c). The curve was fitted by: log transformed number of eggs = 0.0039×female fork length +4.22 for first mating (95 % confidence interval (CI) for a coefficient ranging from 0.0035 to 0.0043 , *p* < 0.01: a), log transformed number of eggs = 0.0044×female fork length +3.82 for second mating (95 % CI for a coefficient ranging from 0.0040 to 0.0048 , *p* < 0.001: b), log transformed number of eggs = 0.0037×female fork length +3.93 for third mating (95 % CI for a coefficient ranging from 0.0032 to 0.0042, *p* < 0.001: c), respectively.

Movie S1. Movie at the moment of gamete release, where male was attached with the condom instrument.

| Table S1　General linear mixed model examining the effects of dependent variables on the estimated number of egg released. A likelihood ratio test was used to the significance of the fixed effects compared with null model. AIC; Akaike's information criteria; CI: 95 % confidence interval for a coefficient; SE; standard error of a coefficient | | | | | | | | |
| --- | --- | --- | --- | --- | --- | --- | --- | --- |
| Dependent variables |  | Estimates |  |  |  |  |  |  |
|  |  | AIC | Log-likelihood | Coefficient | CI for a coefficient | | SE | P-*value* |
|  |  |  |  |  | Lower | Upper |  |  |
| Null |  | 352.4 | -173.2 | - | - | - | - | - |
| Female fork length |  | 351.8 | -171.9 | 0.07 | 0.02 | 0.11 | 0.02 | 0.003 |
| Log transformed duration between matings |  | 343.7 | -167.8 | 14.57 | 2.22 | 26.47 | 6.13 | 0.020 |
| Female fork length + Log transformed duration between matings |  | 344.4 | -167.2 | 0.06 | 0.02 | 0.10 | 0.02 | 0.001 |
|  |  |  |  | 12.28 | 0.49 | 23.99 | 5.91 |  |

| Table S2　Linear mixed model examining the effects of dependent variables on the duration between matings. The duration between matings was log transformed. The pair identity was treated as a random effect. A likelihood ratio test was used to the significance of the fixed effects compared with null model. AIC; Akaike's information criteria; CI: 95 % confidence interval for a coefficient; SE; standard error of a coefficient. | | | | | | | | |
| --- | --- | --- | --- | --- | --- | --- | --- | --- |
| Dependent variables |  | Estimates |  |  |  |  |  |  |
|  |  | AIC | Log-likelihood | Coefficient | CI for a coefficient | | SE | P-*value* |
|  |  |  |  |  | Lower | Upper |  |  |
| Null |  | -36.88 | 21.44 | - | - | - | - | - |
| Female/Male size ratio |  | -37.06 | 22.53 | 0.24 | -0.09 | 0.57 | 0.17 | 0.14 |
| Female fork length |  | -36.40 | 22.20 | 0.0006 | -0.0003 | 0.0014 | 0.0004 | 0.22 |
| Male fork length |  | -35.92 | 21.96 | -0.0004 | -0.0012 | 0.0004 | 0.0004 | 0.31 |
| Estimated amount of sperm released |  | -41.42 | 24.71 | 0.0076 | 0.0014 | 0.0139 | 0.0031 | 0.02 |

| Table S3　Linear mixed model examining the effects of explanatory variables (female fork length) on log transformed number of eggs, egg diameter or egg weight. A likelihood ratio test was used to the significance of the fixed effects compared with null model. AIC; Akaike's information criteria; CI: 95 % confidence interval for a coefficient; SE; standard error of a coefficient | | | | | | | | | | | |
| --- | --- | --- | --- | --- | --- | --- | --- | --- | --- | --- | --- |
| Dependent variables |  | Models | | | | | | | | | |
|  |  | Full model | | | | | |  | Reduced model | | P-value |
|  |  | AIC | Log-likehood | Coefficient | CI for a coefficient | | SE |  | AIC | Log-likehood |  |
|  |  |  |  |  | Lower | Upper |  |  |  |  |  |
| Log transformed number of eggs |  | 2410.3 | -1202.1 | 0.005 | 0.004 | 0.006 | 4.26×10^-4^ |  | 2411.2 | -1202.6 | <0.001 |
| Egg diameter |  | 125.6 | -58.8 | 0.003 | 0.002 | 0.004 | 0.001 |  | 132.1 | -63.0 | <0.001 |
| Egg weight |  | 1484.1 | -738.1 | 0.294 | 0.193 | 0.394 | 0.051 |  | 1507.2 | -750.6 | <0.001 |
